# High Glucose-Induced Senescent Fibroblasts-Derived Exosomal miR-497 Inhibits Wound Healing by Regulating Endothelial Cellular Autophagy via ATG13

**DOI:** 10.1155/ancp/8890200

**Published:** 2025-01-11

**Authors:** Changjiang Liu, Yuting Liu, Yifeng Yu, Siyuan Huang, Chao Sun, Dong Zhang, Aixi Yu

**Affiliations:** ^1^Department of Orthopedics, Zhongnan Hospital of Wuhan University, Wuhan, Hubei 430071, China; ^2^Hubei Clinical Medical Research Center of Trauma and Microsurgery, Wuhan, Hubei 430071, China

**Keywords:** autophagy, diabetic complications, fibroblast, micro-RNA, senescence, wound healing

## Abstract

**Background:** Fibroblasts play a crucial role in diabetic wound healing, and their senescence is the cause of delayed wound repair. It was reported that fibroblasts can secrete exosomes that can mediate a vital role in diabetic complications. Our purpose is to examine the biological function of high glucose (HG)-induced senescent fibroblasts from the perspective of exosomes and reveal the mechanism at cellular and animal levels.

**Methods:** HG-induced senescent fibroblasts were measured by senescence-associated *β*-galactosidase staining and immunofluorescence. Flow cytometry, 5-ethynyl-2′-deoxyuridine (edu), and cell counting kit 8 (CCK-8) assay were applied to detect apoptosis and cell viability. Fibroblasts and endothelial cells were cocultured, and the migration and angiogenesis abilities were detected by scratch, transwell, and tube formation assays. Exosomes were isolated and identified from fibroblasts that were treated differently. Then, the function of exosomes was investigated in cells and mice, including examining the cellular phenotype changes, detecting the autophagy levels, and evaluating the wound healing rate. Furthermore, the potential mechanism by which senescent fibroblast-derived exosomes inhibit wound healing was examined via bioinformatics, real-time quantitive polymerase chain reaction (qPCR), transfection, and dual-luciferase assays.

**Results:** It illustrated that HG-induced senescent fibroblasts exhibited adverse impacts on cellular proliferation, migration, and angiogenesis of endothelial cells via secreting exosomes, and senescent fibroblast-derived exosomes (S-Exos) can delay skin wound defects in mice. Subsequent differential analysis of the GSE153214 and GSE48417 datasets elucidated that miR-497 was the biomarker in the senescent fibroblasts. Interestingly, the miR-497 levels were also elevated in S-Exos. Its overexpression can regulate human umbilical vein endothelial cell function by regulating autophagy via targeting ATG13. Furthermore, *in vivo* experiments also illustrated that miR-497 can delay wound healing and reduce autophagy.

**Conclusions:** Our study demonstrated that exosomes from senescent fibroblasts can impair endothelial cell function and impede diabetic wound healing. The underlying mechanism was that fibroblast-derived exosomal miR-497 can target ATG13 to reduce autophagy, offering insight into new therapy for diabetic complications and other diseases.

## 1. Introduction

The global population with type 2 diabetes mellitus (T2DM) is predicted to increase to 629 million by 2045, with an increasing prevalence among younger individuals [1]. Inadequate management of diabetes can lead to severe complications such as diabetic cardiomyopathy, neuropathy, and nephropathy [2]. Approximately one-third of diabetic patients suffer from skin disorders that can develop into diabetic wounds due to insufficient angiogenesis, leading to delayed or nonhealing wounds [3]. Delayed and nonhealing wound healing in diabetes is often attributed to various hyperglycemia-induced factors, including peripheral nerve and vascular disease, abnormal cellular metabolism and homeostasis, and increased inflammation [4]. Nevertheless, the precise mechanisms underlying diabetic wound healing remain incompletely understood [5].

Delayed-healing diabetic wounds are closely associated with senescence, a state triggered by various stressors, including hyperglymia, oxidative stress, inflammation, and DNA damage [6]. For example, high blood glucose levels in diabetic patients can induce oxidative stress and inflammation, leading to senescence. With aging, the body's anti-inflammatory ability decreases, fostering a chronic low-grade pro-inflammatory state that accelerates the onset of diabetes and its complications, while also hindering tissue repair [7–9]. Mounting studies have highlighted the crucial role of fibroblasts in diabetic wound healing. Delayed-healing diabetic wounds are featured with senescent fibroblasts, which are considered to be the impediment during wound healing [10, 11]. It was demonstrated that phosphorylation of p53 at serine-15 and p21Cip in fibroblast nuclei induced senescence, leading to nonhealing diabetic ulcers [12, 13].

Exosomes are extracellular vesicles with diameters ranging from 50 to 200 nm, released by various cells. These vesicles typically contain miRNA, mRNA, and proteins which can be absorbed by adjacent cells or distant cells through circulation, thereby playing an essential part in intercellular communication [14–16]. Fibroblasts are capable of secreting exosomes and influencing cellular biological processes through miRNA [17, 18]. miRNAs are small noncoding RNA molecules abundant in exosomes, playing a crucial role in wound healing. For instance, miR-20b-5p in exosomes can impair the human umbilical vein endothelial cells (HUVECs) by regulating the Wnt/*β*-catenin signaling pathway and delay diabetic wound healing [19]. Consequently, research on exosomes can provide valuable insights into potential mechanisms and therapeutic strategies.

In our study, we aim to investigate the biological function of high glucose (HG)-induced senescent fibroblasts from the perspective of exosomes and elucidate their role in wound healing. Additionally, we hope to provide new insights into potential therapeutic approaches for diabetic complications and other diseases via exosome research.

## 2. Methods

### 2.1. Cell Culture and Treatment

L929 fibroblasts and HUVECs were purchased from Procell Corporation (Wuhan, Hubei, China). L929 cells were cultured in minimum essential medium (MEM) medium which contained 10% fetal bovine serum (FBS), and HUVECs were cultured in Ham's F-12K medium which contained 10% FBS, heparin (0.1 mg/mL), and endothelial cell growth supplement (ECGs) (0.05 mg/mL). The culture flasks were placed in 95% humidified air and 5% CO_2_. L929 cells were incubated in serum-free medium for 24 h to induce starvation, after which they were assigned to two treatments, i.e., normal glucose (NG; 5.5 mM glucose and 44.5 mM mannitol) and HG (50 mM glucose) conditions [20]. Mannitol was used to eliminate the interference of osmotic pressure on cells. When inhibiting exosome secretion, L929 cells were pretreated with 10 µM GW4869 (MCE, New Jersey, USA). An optical microscope (Perkin Elmer & Olympus, Tokyo, Japan) was used to observe cellular morphology and record the process.

### 2.2. SA-*β*-gal Staining and Immunofluorescence

Cellular senescence was determined by an SA-*β*-gal staining test kit (Beyotime, Shanghai, China). Cells with different treatments were cultured until 80% in six-well plates confluence. Then, cells were immobilized with 4% paraformaldehyde (PFA) for 15 min and incubated with 1 ml SA-*β*-gal staining solution overnight. For immunofluorescence, cells were incubated overnight with p21 (1 : 600, ABclonal, USA) and p16 (1 : 100, ABclonal, USA), after they were fixed and permeabilized.

### 2.3. Edu Assay

The cellular proliferation was detected using the 5-ethynyl-2′-deoxyuridine (edu) Assay kit (Beyotime Biotechnology, China). Cells with different treatments were incubated with the same volume of medium containing Edu solution (10 μM) for 2 h and fixed. The cells were then treated with 0.3% Triton X−100 for 15 min and washed with phosphate buffered saline (PBS) containing 3% bovine serum albumin (BSA). The cellular nuclei were stained with Hoechst 33342. Results were observed and recorded in the optical microscope.

### 2.4. Flow Cytometry Apoptosis Assays

Apoptosis was evaluated according to the Annexin V-Fluorescein isothiocyanate (FITC) apoptosis detection kit (Elabscience, Wuhan, China). After the cells were cultured under different treatments for 48 h, the culture medium and cells were collected. Then, the cell pellets were then washed with PBS. Annexin V binding buffer and V-FITC were added successively for cell incubation. A Cytoflex flow cytometry device (Beckman, USA) was used for detecting differently labeled cells and calculating.

### 2.5. Cell Coculture

To determine the impact of L929 fibroblasts on HUVECs, we performed a coculture experiment. Cells were placed in the upper and lower chambers at a ratio of 1:3 (pore size: 0.4 µm). HUVECs (1.2 × 10^5^ cells/well) and L929 fibroblast cells (0.4 × 10^5^ cells/well) were seeded in the lower and upper chambers, respectively.

### 2.6. Exosome Preparation, Transmission Electron Microscopy, and Particle Size Analysis

The cell culture medium was centrifuged at 4°C and 300 × *g* (10 min), at 2000 × *g* (10 min), and at 10,000 × *g* (30 min). Fluid supernatant was collected and filtered through a 0.22-µm filter, and exosomes were precipitated by ultracentrifugation at 100,000 × *g* (70 min). Then, the precipitated exosomes were washed using PBS and centrifuged again at 100,000 × *g* (70 min). Total protein concentration of exosomes was quantified using a BCA kit (Beyotime Biotechnology). Thereafter, 20 µL of the resuspended sample was added dropwise to 200-mesh grids, which were negatively stained with 2% phosphotungstic acid. Then, we used an HT7800 transmission electron microscope (TEM, HITACHI, Tokyo, Japan) to characterize the samples. For particle size analysis, a nanoparticle tracking analyzer (NTA, Zetaview, Germany) was used to record the diameters of the exosomes.

### 2.7. Exosome Uptake Assay

Uptake assays of HUVECs were performed by labeling exosomes through PKH26 staining (Umibio, Shanghai, China). Cells were cocultured with labeled exosomes for 6 h. The supernatant fluid was then discarded, and cells were sequentially fixed with 4% PFA, followed by 0.2% Triton X-100 incubation for 10 min to permeabilize the membranes, ActinGreen 488 ReadyProbes (Invitrogen, USA) incubation for 30 min, and Hoechst 33342 staining of the nuclei. The results were examined using a laser confocal microscope (Perkin Elmer & Olympus, Tokyo, Japan).

### 2.8. Cell Viability

A cell counting kit 8 (CCK-8 kit) (Elabscience, Wuhan, China) was used to evaluate cell proliferation. N-Exos (Exosomes derived from L929 fibroblasts under NG conditions, 200 μg/mL) and S-Exos (Exosomes derived from senescent L929 fibroblasts under HG conditions, 200 μg/mL) were added to the medium in a 96-well plate, and cells were cultured for 0, 24, 48, 72, and 96 h. Next, 10 µL CCK-8 reagent was added, and we continued to incubate at 37°C (2 h). Absorbance was then measured at 450 nm.

### 2.9. Cell Migration

Cell scratch and transwell assays were conducted to detect cell migration. In the scratch assays, when cells reached 90% confluence, we used a 200-µL tip to create wounds on the surface of the chamber. PBS was used to wash away the detached cells, and different treatments were applied. Images of scratches were observed at 0 and 48 h. In the transwell assay, serum-free cells were seeded into the upper chamber (pore size: 12 µm), while exosomes and other reagents were added to the lower chamber. Cells were fixed with 4% PFA and stained with crystal violet after incubation for 24 h. The migrated cells were examined using an optical microscope.

### 2.10. Angiogenesis

Capillary tube formation assay was used to evaluate angiogenesis ability. In brief, precooled Matrigel (BD Biosciences, San Jose, CA, USA) at 4°C was added to 96-well plates, followed by incubation at 37°C for 30 min. 3 × 10^4^ HUVECs were added to each well and incubated with Matrigel for 6 h. Then, capillary-like structure formation was observed with an optical microscope.

### 2.11. Bioinformatics Analyses

We downloaded the GSE153214 and GSE48417 datasets to identify potential biomarker genes. Both datasets were associated with normal and senescent fibroblasts, of which senescent fibroblasts were induced in GSE48417 and were isolated from aged mice in GSE153214. We used the R package “limma” for data quality control, processing, and differential expression analysis. A Venn diagram was plotted using VeNNY 2.0. Kyoto Encyclopedia of Genes and Genomes (KEGG) pathway analyses were conducted using the miRPath 2.0 software. miRNA target predictions were conducted using seven databases (TargetScan, ENCORI, miRDB, miRWalk, RNA22, RNAInter, and TargetMiner).

### 2.12. Cell Transfection

For cell transfection, HUVECs at the logarithmic growth stage were cultivated in a six-well plate. When cell density reached 30%–50%, we transfected cells with miR-497 mimic (5′-3′: CAGCAGCACACUGUGGUUUGU; 3′-5′: GUCGUCGUGUGACACCAAACA) or mimic NC (5′-3′: UUUGUACUACACAAAAGUACUG; 3′-5′: AAACAUGAUGUGUUUUCAUGAC) (miR10003383-1-5, Ribobio, Guangzhou, China) according to the instruction of a ribs FECT CP Transfection Kit (Ribobio, China), followed by incubation for 24–48 h.

### 2.13. Luciferase Reporter Assay

HUVECs at the logarithmic growth stage (0.5–2 × 10^5^) were seeded into a 24-well plate. When cell density reached 30%–50%, we cotransfected them with dual-luciferase vectors (pGL6-miR-ATG13-WT-3′ UTR, pGL6-miR-ATG13-Mut-3′ UTR) and miR-497 mimics/NC according to Ribo FECT CP transfection kit. The luciferase activity was detected by using the Dual-Luciferase Reporter Assay System (Promega) after 48 h post-transfection.

### 2.14. Quantitative Reverse-Transcription Polymerase Chain Reaction (qRT-PCR)

For qRT-PCR, cells were collected, and total RNA was isolated using an RNA fast200 kit (Feijie, Shanghai, China). RNA concentration was determined using a NanoDrop 2000 device. Then, RNA was reverse-transcribed into complementary DNA. SYBR Green realtime PCR Master Mix (TOYOBO) was used for amplification. Amplification conditions are as follows: incubation at 94°C for 10 min, then 94°C for 5 s, and 60°C for 30 s, for 40 cycles. Taking U6 as the internal control, 2^−*ΔΔ*Ct^ was used to analyze the data. The following primers were used: miR-497-RT (5′-3′): CTCAACTGGTGTCGTGGAGTCGGCAATTCAGTTGAGACAAACCA; miR-497-F, ACACTCCAGCTGGGCAGCAGCACACTGTG.

### 2.15. Western Blotting

For western blotting, RIPA total protein lysate (ASPEN) was utilized for total protein extraction, and a BCA kit (Beyotime) was used to detect protein concentrations. Proteins were separated using 10% or 15% sodium dodecyl sulfate-polyacrylamide gel electrophoresis. Then, they were transferred to polyvinylidene fluoride membranes (Merck Millipore, Burlington, MA, USA) and blocked with 5% skim milk for 1.5 h at room temperature. Then, the membranes were incubated with primary antibodies at 4°C overnight and washed by Tris-buffered saline (TBST (150 mM NaCl, 10 mM Tris, pH 7.4) containing 0.1% Tween) three times. Next, the membranes were incubated with horseradish peroxidase (HRP)-conjugated secondary antibodies at 37°C for 1 h and washed. Finally, an enhanced chemiluminescence assay (ECL, Biosharp) was used to visualize. The primary antibodies mainly included *β*-actin (1 : 10,000, Abcam), GAPDH (1 : 10,000, Abcam), p16 (1 : 2000, Abcam), p21 (1 : 1000, Abcam), vascular endothelial growth factor (VEGF) (1 : 2000, Abcam), CD31 (1 : 1000, Abcam), ATG13 (1 : 1000, abclonal, USA), and LC3B (1 : 1000, cell signal, USA).

### 2.16. Animal Model

Male BALB/c mice (8 weeks old) were purchased from the Wuhan University Animal Center (Ethical number: ZN2023151). Diabetes was induced in mice by being injected with streptozotocin (STZ, 60 mg/kg, Solarbia) intraperitoneally. The mice were anesthetized with 1% pentobarbital sodium (Sigma–Aldrich, St. Louis, MO, USA), and hair in the surgical area on the back was shaved. A circular full-thickness skin lesion (8 mm diameter) was inflicted aseptically. The mice were randomized and subcutaneously injected with exosomes or PBS at four injection sites (25 µL at each site; 1 µg/µL). For mimic-NC and mimic-miR-497, we ensured the use of an equal volume of Lipofectamine 3000 (Thermo Fisher) with a dose of 2 nmol per wound. Wound changes were observed 0, 3, 7, and 10 days after surgery. ImageJ 6 software (Media Cybernetics, Bethesda, MD, USA) was used to calculate the wound area. The wound closure rate was calculated: wound closure rate = (*A*_0_–*A*_t_)/*A*_0_ × 100%, where *A*_0_ was the initial wound area, and *A*_t_ was the wound area on days 3, 7, or 10 post-surgery. The mice were sacrificed 10 days after surgery, and wound tissues were collected.

### 2.17. Histological Staining

Wound tissues were fixed with 4% PFA and then embedded into paraffin. A Leica RM2016 electric microtome was used to section the tissues. Hematoxylin and eosin (H&E) staining was performed for general tissue observation, while Masson's trichrome staining was used to assess collagen deposition. CD31 immunofluorescence (1 : 1000, Abcam, Cambridge, UK) was used for angiogenesis analysis. For electron microscopy, skin tissues were fixed in 3% glutaraldehyde with 0.1 M phosphate buffer (pH 7.4), and post-fixed in 1% osmium tetroxide for 2 h. After dehydration, infiltration, and embedding, samples were cut sectioned and dyed.

### 2.18. Statistical Analyses

Data are shown as means ± standard deviation of at least three separate experiments. Analyses were performed using R 3.6.3 software and GraphPad Prism 7 (San Diego, CA, USA). The *P*-values were adjusted following the Benjamini–Hochberg method. Student's *t*-test was used to evaluate differences between the two groups. Statistical significance is reported at *p* < 0.05. n.s. = not statistically significant, *⁣*^*∗*^*p* < 0.05, *⁣*^*∗∗*^*p* < 0.01, *⁣*^*∗∗∗*^*p* < 0.001.

## 3. Results

### 3.1. Induced Senescent Fibroblasts in HG Conditions

**Figure 1:**
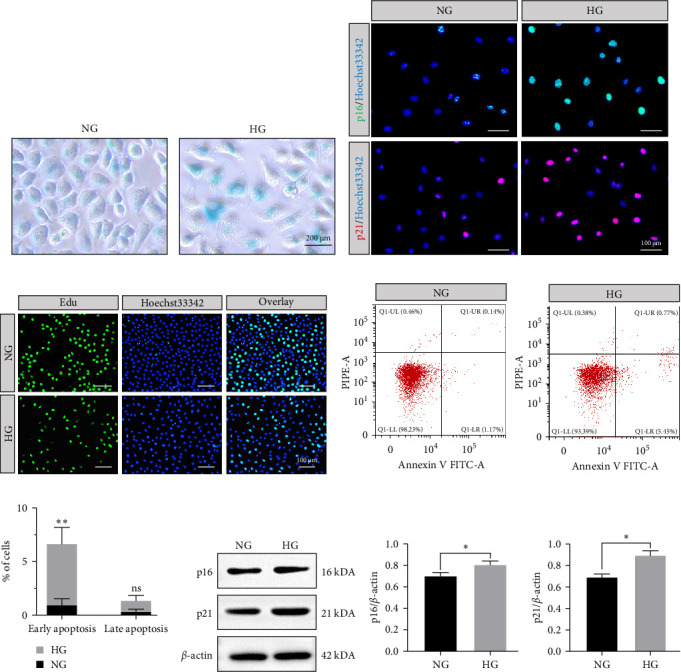
Induced senescent L929 fibroblasts under high glucose (HG) condition. (A) SA-*β*-gal staining of L929. (B) Immunofluorescence of L929. (C) Edu proliferation assays of L929. (D, E) Apoptosis in L929 fibroblasts was detected by flow cytometry. (F–H) The protein levels of p16 and p21 in L929 under the NG and HG treatments. *⁣*^*∗*^*p* < 0.05 and *⁣*^*∗∗*^*p* < 0.01. NG, normal glucose.

We compared the effects of different glucose concentrations (5.5 and 50 mM) on L929 fibroblasts. HG treatment increased senescence-associated *β*-galactosidase staining (Figure 1a) and elevated the protein levels of p16 and p21 (Figure 1b). Additionally, cell proliferation in the HG group was significantly reduced (Figure 1c). Fibroblasts in the HG group indicated a higher early apoptosis index (Figure 1d,e). Besides, western blotting results elucidated that protein levels of p16 and p21, which were highly related to senescence, were elevated in the HG group (Figure 1f–h).

### 3.2. Senescent Fibroblasts Inhibit HUVEC Functions via Secreting Exosomes

**Figure 2:**
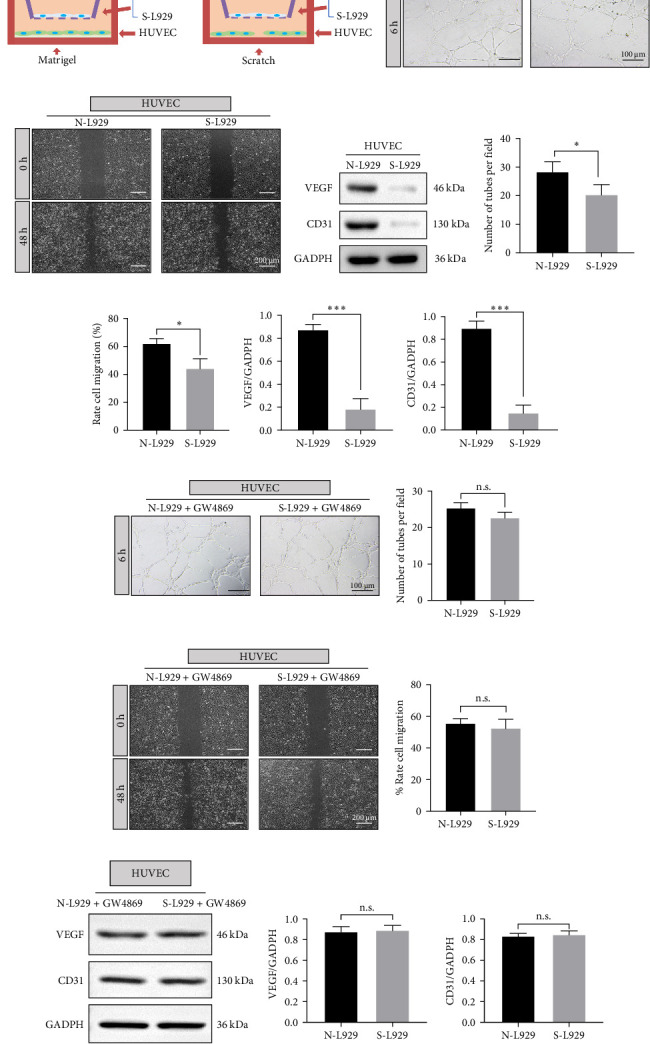
Coculture of L929 fibroblasts and HUVECs. (A) Diagram of coculture system. (B) Tube formation of HUVECs when coculture with L929 in 6 h. (C) Scratch assay of HUVECs in 0 and 48 h. (D) Protein levels of VEGF and CD31 in HUVECs. (E, F) Quantitative analysis of tube formation and cell migration. (G, H) Quantitative analysis of VEGF and CD31 protein levels. (I) Tube formation by HUVECs when added to GW4869. (J) Scratch assay of HUVECs when added to GW4869. (K) Protein levels of VEGF and CD31 in HUVECs when added to GW4869. *⁣*^*∗*^*p* < 0.05 and *⁣*^*∗∗∗*^*p* < 0.001. HUVEC, human umbilical vein endothelial cell; VEGF, vascular endothelial growth factor.

The coculture system was shown in Figure 2a. Coculture with senescent fibroblasts reduced tube formation (Figure 2b,e) and migration ability (Figure 2c,f) of HUVECs. Additionally, the protein levels of VEGF and CD31 in the S-L929 group were substantially declined (Figure 2d,g,h). Furthermore, GW4869 can inhibit exosome secretion. After adding GW4869, the tube formation and migration ability of HUVECs in the S-L929 group became similar to those in the N-L929 group (Figure 2i,j). In addition, the protein levels of VEGF and CD31 in the two groups were very close (Figure 2k). We assumed that exosomes might play an essential role in cell interaction between fibroblasts and endothelial cells. Besides, exosomes derived from senescent fibroblasts may exhibit harmful effects on endothelial cells.

### 3.3. Identification of Exosomes

**Figure 3:**
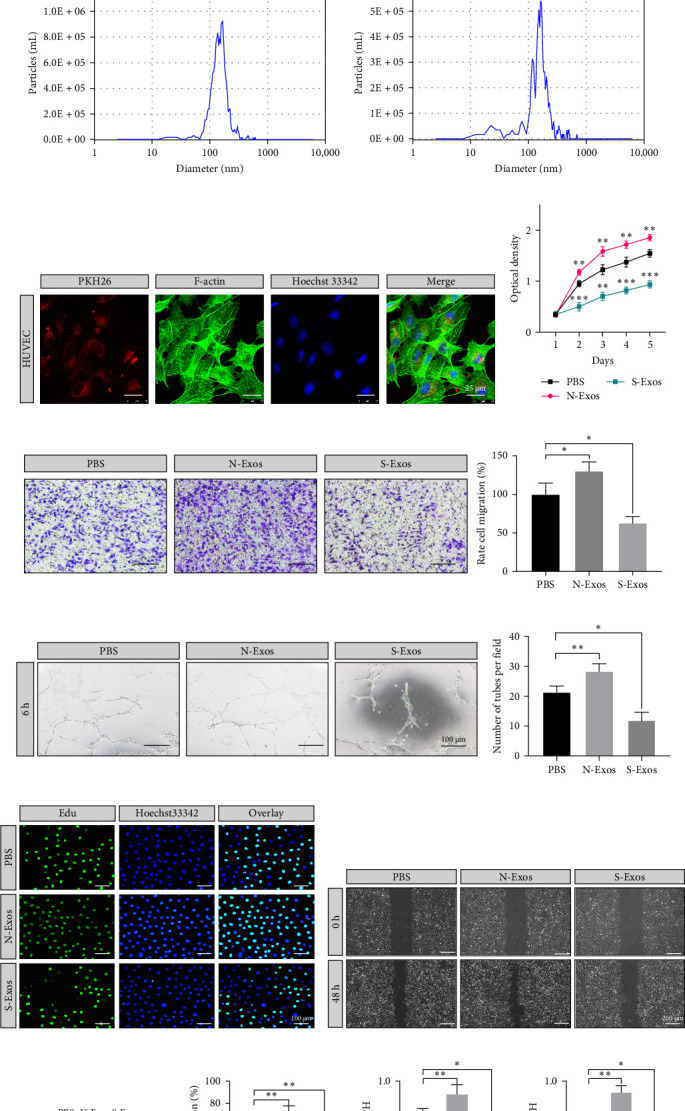
Exosome Characterization. (A) Exosome morphology observed by TEM. (B) Confirmation of exosomal biomarkers of CD9, CD81, and TSG101. (C, D) Particle size and distribution of N-Exos and S-Exos, respectively. (E) Exosomes uptake assay. (F) Cell proliferation curves when treated with PBS, N-Exos, and S-Exos. (G) Transwell assays of HUVECs migration ability. (H) Tube formation of HUVECs. (I) Edu proliferation assays of HUVECs. (J) Scratch assays of HUVECs migration ability. (K) Western blotting of VEGF and CD31 in HUVECs. (L) Quantitative analysis of scratch assays. (M) and (N) Quantitative analysis of protein levels of VEGF and CD31. *⁣*^*∗*^*p* < 0.05, *⁣*^*∗∗*^*p* < 0.01, and *⁣*^*∗∗∗*^*p* < 0.001. HUVEC, human umbilical vein endothelial cell; PBS, phosphate buffered saline; TEM, transmission transmission electron microscope; VEGF, vascular endothelial growth factor.

According to the mentioned procedures, fibroblast-derived exosomes were isolated and named N-Exos and S-Exos, which were derived from normal L929 and senescent L929, respectively. TEM images showed that the exosomes exhibited typical exosomal structures, including spherical, homogeneous, and membrane vesicles (Figure 3a). Exosomal-specific proteins (CD9, CD81, TSG101) were identified by western blotting (Figure 3b). The NTA confirmed that the average diameter of N-Exos was 167.5 ± 64.3 nm, and that of S-Exos was 186.7 ± 90.2 nm (Figure 3c,d). Furthermore, fluorescence microscopy images showed that these PKH26-labeled exosomes could be endocytosed by HUVECs (Figure 3e).

### 3.4. S-Exos Inhibited Cellular Proliferation, Migration, and Angiogenesis In Vitro

We performed CCK-8 and edu assays to explore cellular proliferation. Transwell and scratch assays were used to evaluate migration ability. A tube formation assay was used to assess angiogenesis. S-Exos decreased the proliferation rate of HUVECs, whereas N-Exos promoted it (Figure 3f). Transwell assays elucidated that HUVECs treated with S-Exos displayed a worse migration rate than that in PBS and N-Exos groups (Figure 3g). Angiogenesis ability was also damaged in S-Exos (Figure 3h). The ratio of edu-positive cells was lower in the S-Exos group (Figure 3i). Besides, at 48 h, less transverse migration can be observed in the S-Exos group (Figure 3j,l). The protein levels of VEGF and CD31 were both reduced in S-Exos group compared to the PBS and N-Exos groups (Figure 3k,m,n).

### 3.5. S-Exos Inhibited Wound Healing in Mice

**Figure 4:**
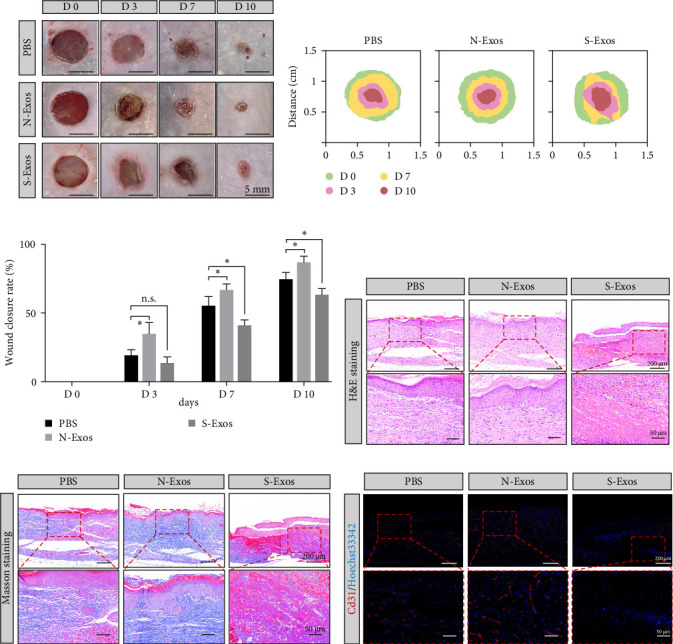
The functions of exosomes in wound healing of mice. (A) Representative images of full-thickness skin defect in mice treated with PBS, N-Exos, and S-Exos. (B) Diagram of wound traces. (C) Changes in the wound area during healing. (D, E) HE and Masson staining of wound tissues, respectively (×15 and ×45). (F) Immunofluorescence of CD31 in three groups. *⁣*^*∗*^*p* < 0.05. PBS, phosphate buffered saline.

The effect of S-Exos on wound healing in normal mice was investigated. Representative wounds at various time points were shown in Figure 4a. On day 7, wound healing was significantly reduced in three groups. However, the wound area in the S-Exos group was wider than that in the PBS and N-Exos groups. S-Exos exhibited the poorest therapeutic effect. On day 10, S-Exos-treated wound healing was delayed (Figure 4b). Wound closure rates also validated the overall results (Figure 4c), with no difference between the S-Exos and PBS groups on day 3. From day 7 onward, the wound healing rate in the S-Exos group was significantly lower than that in the PBS group, while N-Exos treatment promoted healing. H&E staining results are shown in Figure 4d. Masson's trichrome staining indicated less collagen and a higher degree of disorder in the S-Exos group (Figure 4e). Immunofluorescence analysis showed that blood vessel density was highest in the N-Exos group, while the number of regenerated blood vessels and vessel density were lower in the S-Exos group compared to the PBS group (Figure 4f).

### 3.6. miR-497 Mediated Senescence and Its Function in HUVECs

**Figure 5:**
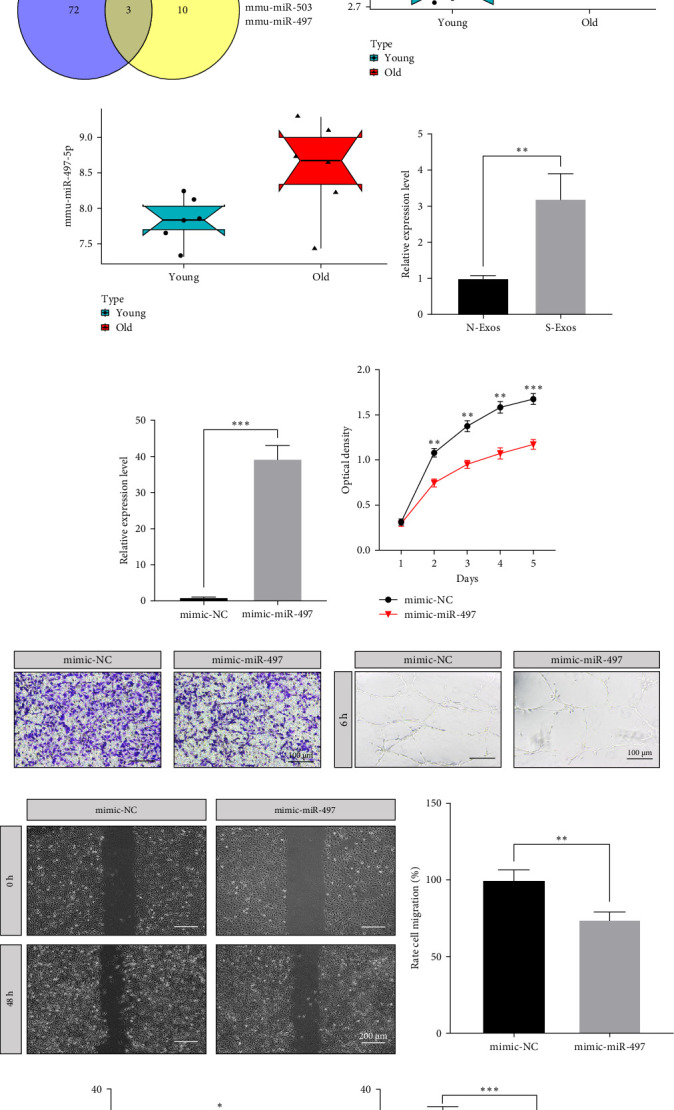
Role of miR-497 in senescence and its function in HUVECs. (A, B) Heatmap of differentially expressed genes in GSE143214 and GSE48417. (C) Venn diagram of intersecting genes. (D, E) Boxplots of the expression level of miR-497 in GSE143214 and GSE48417, respectively. (F) Levels of miR-497 in N-Exos and S-Exos by qPCR. (G) Levels of miR-497 in HUVECs transfected by mimic-NC and mimic-miR-497. (H) Cell proliferation curves of HUVECs in two groups. (I) Transwell assays of HUVECs. (J) Tube formation of HUVECs. (K) Scratch assays of HUVECs. (L), (M), and (N) Quantitative analysis of transwell, tube formation, and scratch assays, respectively. (O) The edu proliferation assay of HUVECs in mimic-NC and mimic-miR-497 groups. (P) The protein levels and quantitative analysis of VEGF and CD31 in HUVECs. *⁣*^*∗*^*p* < 0.05, *⁣*^*∗∗*^*p* < 0.01, and *⁣*^*∗∗∗*^*p* < 0.001. HUVEC, human umbilical vein endothelial cell; qPCR, real-time quantitive polymerase chain reaction; VEGF, vascular endothelial growth factor.

We downloaded two GEO datasets that were related to fibroblast senescence. After data processing, quality control, and differential expression analyses, 75 and 13 miRNAs were identified in GSE153214 and GSE48417, respectively. Heatmap of two matrixes were drawn in Figure 5a,b. We then identified three intersecting miRNAs (miR-130b, miR-503, and miR-497) (Figure 5c). However, the expression levels of miR-130b and miR-503 were inversely correlated between young and old groups. miR-497 was upregulated in old fibroblasts in GSE153214 and GSE48417, respectively (Figure 5d,e). Additionally, the expression level of miR-497 was elevated in S-Exos (Figure 5f). We then investigated whether miR-497 participated in regulating the function of HUVECs. Then, HUVECs were then transfected with mimic-miR-497 and mimic-NC (Figure 5g) and detected by real-time quantitive polymerase chain reaction (qPCR). CCK-8 assays showed that HUVECs transfected with mimic-497 displayed a slower proliferation rate (Figure 5h). miR-497 overexpression inhibited the vertical migration of HUVECs (Figure 5i,l), as well as the angiogenesis ability (Figure 5j,m). Scratch assays demonstrated that the transverse migration ability of HUVECs was also impaired in the mimic-miR-497 group (Figure 5k,n). The number of edu-positive HUVECs in the mimic-497 groups was lower than that in the mimic-NC group (Figure 5o). The protein levels of VEGF and CD31 were also reduced in the mimic-497 group (Figure 5p).

### 3.7. miR-497 Impaired HUVECs Function by Regulating Autophagy Level via Targeting ATG13

**Figure 6:**
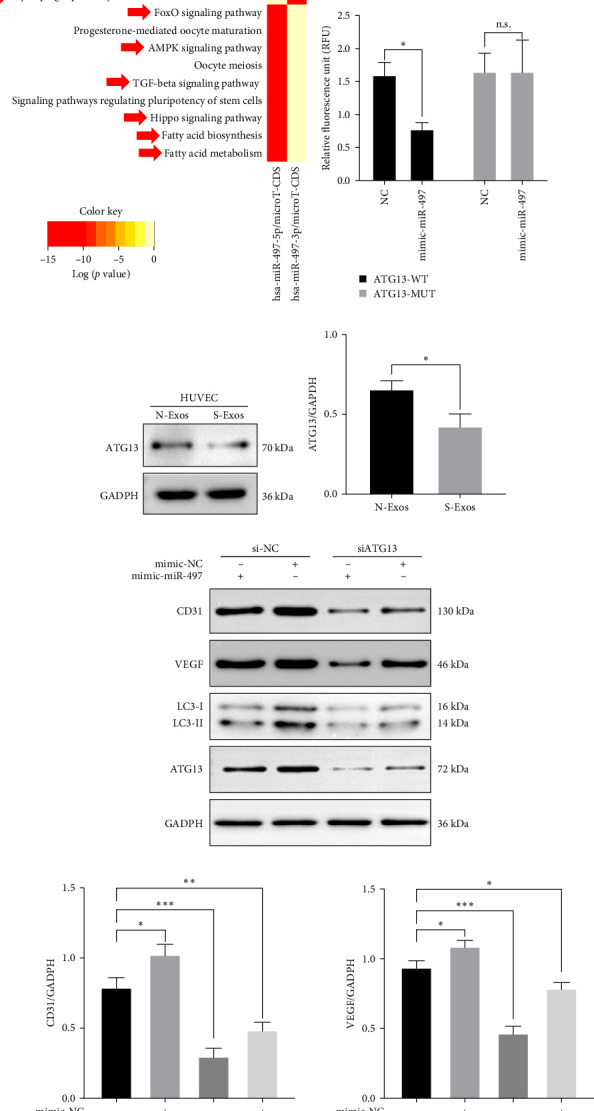
miR-497 impaired HUVECs function by regulating autophagy level via targeting ATG13. (A) Venn diagram of miR-497 targeted genes in seven databases. (B, C) Functional analysis of miR-497 in two databases by miRpath2.0 software. (D) Reciprocal mapping and double luciferase assay results. (E, F) The protein levels of ATG13 in HUVECs when treated with N-Exos and S-Exos. (G) and (H) The protein levels of CD31, VEGF, LC3-II/I, and ATG13 in HUVECs when cotransected by mimic-miR-497 or siATG13. *⁣*^*∗*^*p* < 0.05, *⁣*^*∗∗*^*p* < 0.01, and *⁣*^*∗∗∗*^*p* < 0.001. HUVEC, human umbilical vein endothelial cell; VEGF, vascular endothelial growth factor.

Subsequently, to examine the mechanism by which miR-497 regulated the HUVECs, seven bioinformatics tools were utilized to identify putative targets, and 448 genes were predicted (Figure 6a). We also enriched the potential pathways of miR-497 (Figure 6b,c). miR-497 was found to be associated with autophagy-related functions (e.g., mTOR and FoxO signaling pathways), immune-related functions (e.g., TGF-beta signaling), metabolism-related functions, and other classical pathways. Among these candidate target genes, ATG13 played a key role in the autophagy process and was eventually selected as the target gene for further investigation. Then, we conducted a luciferase reporter assay to identify the interactions between miR-497 and 3′-UTR of ATG13 in HUVECs. As shown in Figure 6d, overexpression of miR-497 significantly suppressed the luciferase activity of ATG13-WT, whereas no difference was observed in the ATG13-Mut group. Then, we also detected the ATG13 protein level in HUVECs treated with N-Exos or S-Exos. It was illustrated that ATG13 decreased in the S-Exos group (Figure 6e,f). Furthermore, HUVECs were cotransfected with mimic-miR-497 and mimic-NC, and the angiogenesis-related and autophagy-related protein levels were determined. The results showed that the protein levels of CD31, VEGF, LC3-II/LC3-I, and ATG13 were reduced by mimic-miR-497, and si-ATG13 further aggravated their decrease and downregulated autophagy levels (Figure 6g, h). These results suggested that miR-497 can regulate HUVEC function and autophagy levels through targeting ATG13.

### 3.8. miR-497 Impaired Autophagy and Wound Healing in Diabetic Mice

**Figure 7:**
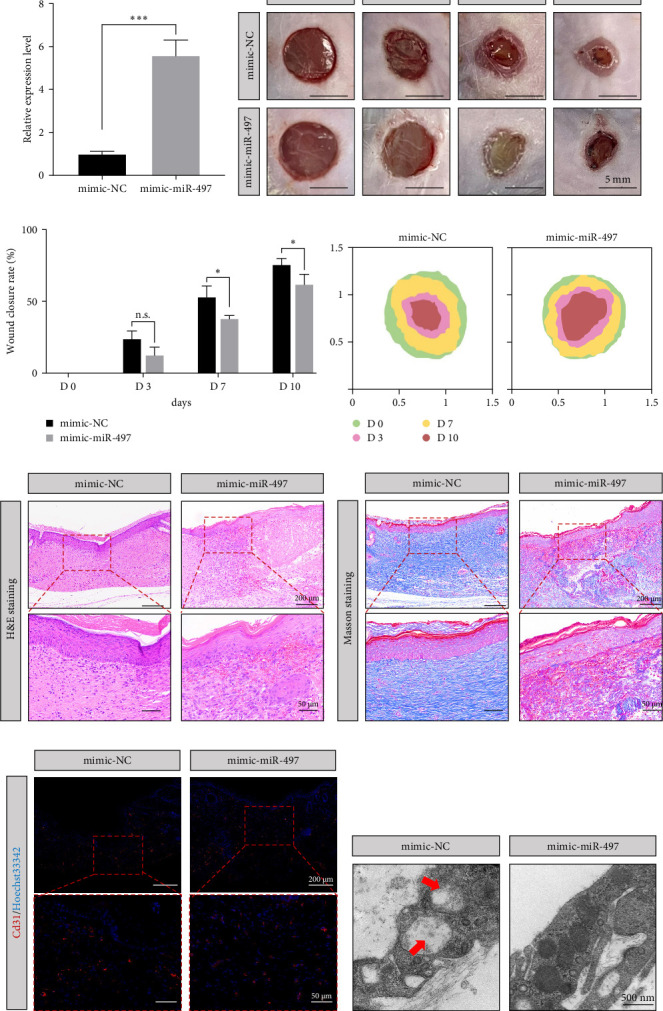
Functions of mimic-miR-497 in wound healing. (A) Levels of miR-497 in two groups by qPCR. (B) Representative images of full-thickness skin defects in mice treated by mimic-NC and mimic-miR-497. (C) The wound area changes during healing. (D) Wound traces diagram. (E, F) HE and Masson staining of wound tissues, respectively (×15 and ×45). (G) CD31 immunofluorescence in two groups. (H) Histological electron microscopy results. *⁣*^*∗*^*p* < 0.05 and *⁣*^*∗∗∗*^*p* < 0.001. qPCR, real-time quantitive polymerase chain reaction.

The effects of mimic-miR-497 were investigated in diabetic mice. The expression level of miR-497 at the wound site was detected by qPCR at 24 h after injection. It was found that the expression level of miR-497 in the mimic-miR-497 group was significantly higher than that of the mimic-NC group, indicating effective delivery (Figure 7a). Representative wounds at various time points are shown in Figure 7b. The wound closure rate in the mimic-miR-497 group was lower than that in the mimic-NC group (Figure 7c). On day 10, the wound area in the mimic-miR-497 group was wider than that in the mimic-NC group (Figure 7d). H&E staining and Masson's trichrome staining showed increased inflammatory infiltration and reduced collagen accumulation in the mimic-miR-497 group (Figure 7e,f). Immunofluorescence showed that blood vessel density in the mimic-miR-497 group was less than the control group (Figure 7g). TEM analysis demonstrated reduced autophagy in the mimic-miR-497 group (Figure 7h).

## 4. Discussion

Fibroblasts play a crucial role in wound healing. Upon tissue damage, fibroblasts become activated and migrate to the wound site. However, in diabetic wounds, fibroblasts are found to be senescent [12, 21, 22]. HG is a primary factor in skin senescence and functional disorders [23–25], strongly associating HG conditions with cellular senescence and potentially contributing to delayed wound healing. Our purpose is mainly to examine the biological function of HG-treated senescent fibroblasts from the perspective of exosomes and reveal the mechanism of regulating endothelial cells in wound healing.

Firstly, we induced senescent fibroblasts by HG treatment and evaluated their biological effects on HUVECs. HG condition can increase the SA-*β*-gal activity and expressed levels of p16 and p21. Cell proliferation was reduced, and apoptosis was increased in senescent fibroblasts. Then, we cocultured senescent fibroblasts with HUVECs and found that they impaired the HUVECs in migration and angiogenesis ability. The addition of an exosome inhibitor alleviated the effects of senescent fibroblasts on HUVECs, indicating that exosomes play a key role in cellular communication. Next, we isolated and identified S-Exos and N-Exos and examined their effects, where S-Exos impaired the function of HUVECs. *In vivo* experiments also showed that S-Exos delayed wound healing and inhibited angiogenesis.

Furthermore, to explore the potential mechanisms of exosomes derived from senescent fibroblasts, we downloaded two GEO datasets (GSE153214 and GSE48417) to screen biomarkers associated with senescent fibroblasts. miR-497 was identified as a potential miRNA, and we confirmed that it was upregulated in S-Exos. Then, we transfected mimic-miR-497 into HUVECs to examine its function. It was found that overexpressed miR-497 impaired HUVEC function. miR-497 was associated with several biological processes, including autophagy and inflammation. We then predicted its target genes using seven databases and identified overlapping targets. ATG13 was a candidate gene and downregulated in HUVECs treated with S-Exos. A luciferase reporter assay illustrated that in HUVECs, overexpression of miR-497 can significantly decrease the luciferase activity of ATG13-WT, whereas no difference in the ATG13-Mut group. miR-497 can regulate autophagy levels via mediating ATG13. In diabetic mice, we found miR-497 delayed wound healing and reduced autophagy. Therefore, miR-497 was identified as a crucial biomarker in S-Exos for regulating autophagy during the process of wound healing. It revealed the potential for regulating miR-497 to facilitate diabetic wound healing.

Autophagy and senescence are interconnected cellular processes that play important roles in maintaining organism homeostasis [26, 27], including in diabetes [28, 29]. Targeting cellular senescence and enhancing autophagy have emerged as potential therapeutic strategies for improving diabetic wound healing. miR-497 is a microRNA that plays an essential role in various biological processes. It was regarded as a tumor suppressor gene, including inducing apoptosis, inhibiting proliferation, and suppressing migration and invasion in multiple types of cancer [30–32]. In nontumor diseases, overexpression of miR-497 can restrain blood vessel formation via regulating downstream STAT3 and VEGFA expression [33]. The relationship between miR-497 and autophagy has not been widely studied in skin and wound healing yet. However, in neurodegenerative disorders, some studies elucidated that the silencing of miR-497 can inhibit cell apoptosis and promote autophagy [34, 35]. In our study, we identified ATG13 as a target gene of miR-497 and found that overexpression of miR-497 derived from senescent fibroblast exosomes regulates autophagy in HUVECs by targeting ATG13. Furthermore, we confirmed that miR-497 ameliorated autophagy during wound healing in diabetic mice. These results indicate that miR-497 derived from senescent fibroblasts impairs endothelial cell function and delays wound healing by regulating autophagy through downregulating ATG13 expression.

## 5. Conclusions

In this work, we explored the biological correlation between HG-treated senescent fibroblasts and endothelial cells in wound healing. Senescent fibroblast-derived exosomes can contribute to delayed diabetic wound healing. Additionally, miR-497 was identified as a biomarker in senescent fibroblasts and their exosomes. Over-expression of miR-497 in S-Exos impaired endothelial cells and reduced the autophagy level by targeting ATG13, thereby hindering wound healing. These findings provide new insights into the mechanisms of delayed wound healing in diabetes and suggest potential new therapies for wound healing and other related diseases.

## Data Availability

The datasets used and/or analyzed in the current study are available from the corresponding author upon reasonable request.

## Nomenclature

T2DM: Type 2 diabetes mellitus
HG: High glucose
S-Exos: Senescent fibroblast-derived exosomes
N-Exos: Normal fibroblast-derived exosomes
SA-*β*-gal staining: Senescence-associated *β*-galactosidase staining
HUVECs: Human umbilical vein endothelial cells
Edu: 5-Ethynyl-2′-deoxyuridine
CCK-8: Cell counting kit.
